# Medical Items and News of Interest to the Physician

**Published:** 1884-10

**Authors:** 


### Medical Items and News,

### For the Use of Physicians.

CULLED FROM THE STANDARD MEDICAL JOURNALS OF THE WORLD.

NOTE.—These formulæ are intended only for the regular practitioner of medicine, and should be employed only by him, or under his immediate supervision.

**Eczema.**

Carbonate of lead, 2 drachms; sulphate of morphia, 10 grains; chloroform, 2 drachms; glycerine, 2 ounces. Mix. For external use in eczema rubrum.—*St. Louis Med. Journal.*

**Bromidia.**

Dr. J. T. Larew, Prof. Minor Surgery, St. Louis Col. Phy. and Sur., says: "In my opinion Bromidia is a safe and effective hypnotic, far superior to any of its ingredients alone or to the combination as prepared from the ordinary drugs."

**Diarrhœa in Infants.**

Dr. Lewin has found great success in the treatment of diarrhœa in young infants by giving them soluble albuminate of tannin, made by adding the white of an egg to a solution of tannin; The white of egg must be previously beaten up with some water.—*Ther. Gazette.*

**Piles.**

R

Iron sub. sulphate, gr. xxx.
Morphia sulphate, gr. ij.
Iodoform, gr. iij.
Cacao butter, gr. 165

Make into ten suppositories.—*The Druggist.*

**Pruritus Vulvæ.**

Dr. William Goodell recommends for pruritus vulvæ:

R

Carbolic acid, one drachm.
Morphia sulphate, ten grains.
Boracic acid, two drachms.
Vaseline, two ounces. M.

Also, pat the parts with a sponge soaked in boiling-hot water. This is also a most excellent application for that rawness so often found between the thighs of the newly born.—*Medical Herald.* [What, with ten grains of morphine? Guess you had better not try it on the "newly born."—ED. BISTOURY.]

**Cholera Infantum, Summer Diarrhœa, etc.**

Dr. Bartholow uses

R

Bismuth subnit, grs. lx.
Sach. pepsin, grs. xxx.
Zinci oxidi, grs. vj.

Mix.—Divide into 12 powders. Sig.—One powder every 4 to 6 hours.—*Medical World.*

**Scarlatina.**

Dr. James Couldrey, in the *Lancet*, writes: "In an epidemic of scarlatina of a severe character I have gotten very great benefit in several cases by the prompt exhibition of salicylate of soda in doses of fifteen grains every two hours to adults, until singing in the ears is produced, and then every four hours during the first week.—*Louisville Med. News.*

**Seat Worms.**

Dr. F. A. Jordan, of Pecatonica, Ill., states that he has cured a case of seat worms (ascaris vermicularis) of over twenty years' standing, in which the patient frequently passed hundreds of worms, by the use of half teaspoonful doses of fluid extract of cascara sagrada every night at bed-time. The cure has now lasted six months. Probably the use of the same remedy as an enema would also be efficacious.

**Cancer.**

M. Molliere maintains, in *Lyon Medicale*, that the internal administration of five or six grams (from 75 to 90 grains) of the salicylate of soda will relieve the pain of cancer of the uterus, even after morphine has been given without effect. He says nothing of the effect of this salt upon the pain of cancer as it occurs in other tissues, but the reader will doubtless act on the suggestion and not confine its use to the disease as it affects the uterus.

**Gout.**

Dr. N. S. Davis, of Chicago, recommends forty drops of an equal mixture of the acetated tincture of opium and wine of colchicum seeds to control acute paroxysms of gout. This dose may be repeated in an hour if necessary. Oftentimes, one or two doses will abort what threatens to be a very severe attack. When the paroxysm is under control the same remedies may be continued in smaller doses, three or four times daily, if any gout remains. We have used this remedy, and can add our indorsement to this distinguished recommendation.—*Medical and Surgical Reporter.*

**Epidemic Diseases.**

Mr. Burq, after a statistical research, thinks that there is almost absolute immunity to copper workers in epidemics of cholera and typhoid fever. The statistics which he has collected seem to show that the storing up of copper, in small amounts, in the animal economy constitutes an almost infallible protection.

---

**Freckles.**

Dr. J. V. Shoemaker, of Philadelphia, states as the result of a somewhat extensive experience, that the careful application of a small piece of the ointment of the oleate of copper at night will usually remove freckles. The oleate of copper ointment is prepared by dissolving one drachm of the salt in a sufficient quantity of oleo-palmitic acid to make a soft ointment.

---

**Gonorrhœa.**

The *Deutsches Archiv fur Klin. Med.* has been carefully reading reports as they have appeared in American, German, French and English medical journals, of the action of the yellow oil of sandal wood upon specific urethritis. As a result it recommends very highly the exhibition of from 15 to 20 drops of this oil three times a day, and gives preference to its administration in gelatine capsules.

---

**Freckles.**

℞

Oil of almonds, exp'd, fl. ℥ iv,
Lard, ℥ iij,
Spermaceti, ℥ j,
Expressed juice of house leek, fl. ℥ iij.

Melt the spermaceti and lard together; add the oil and then the juice and stir the mixture until it solidifies on cooling. A few drops of some perfume, as cologne, may be added — *Oil and Drug News.*

---

**Facial Erysipelas.**

In simple cases of facial erysipelas, Professor Bartholow has seen the most gratifying results from the employment of belladonna. Where there is much systematic depression quinine should be given in a pill, every four hours, composed of the following: Extract of belladonnæ, one-quarter of a grain, and sulphate of quinine three grains. Locally, vaseline, lard, or a weak mercurial ointment may be applied. This, with a cooling laxative, will geneaally complete the cure in a few days.—*The Medical Bulletin Advertiser.*

**Belladonna.**

Minim doses given every half hour is a good remedy in nasal catarrh and bronchitis, accompanied with free secretion. Owing to its tendency to cumulative effects, it should be suspended for a time after eight or ten doses are given. Thus administered, it retards the exudation of serum and sustains the heart's action in œdema.—*Weekly Med. Rev.*

---

**Liniment for Weak Back.**

P. M. Dromgold, M D., uses the following:

℞

Tr. cantharides.
Tr. camphor, aa ℥ iv.
Oil of cedar, ℥ j.
Turpentine.
Aquæ ammonia, aa ℥ iv.

Mix as above given.—*Med. World.*

---

**Beck's Expectorant Mixture.**

℞

Hydrochlorate of apomorphine, grs. jss.
Dilute hydrochloric acid gr. xxx.
Simple syrup, ℥ ii
Distilled water, ℥ vi. M.

Dose.—For an adult, one tablespoonful; for a child, one teaspoonful every two or three hours.—*Jour. de Med. de Paris.*

---

**Diet in Diabetes.**

Prof. William Pepper, of the University of Pennsylvania, recommends the following bill of fare in glycosuria, to be replaced in certain cases by a skimmed milk diet:

Breakfrst—A cup of tea, without milk or sugar, but with a slice of lemon in it, according to the Russian fashion; a couple of soft-boiled eggs, broiled chops, beefsteak or fish; oysters must be excluded, as the liver, which makes up the chief bulk of the oyster, contains sugar; with these may be eaten some vegetable, as a raw tomato, a raw onion with vinegar, and a slice of gluten bread, or a couple of gluten biscuits.

Between breakfast and dinner a little cream, with a teaspoonful or old rum or whiskey.

Dinner—Meat, green vegetables, string beans, tomatoes, cauliflower, onion, lettuce (the latter contains a little sugar, but not enough to do any harm), and again gluten bread. The evening meal is similar to the breakfast.—*Med. Record.*

**Dysentery.**

℞

Magnesia Sulph, ʒ iv.

Syr. Ipecac, ʒ iv.

Tinct. Opii Camph., ʒ iv.

Tinct. Cinnamonia, ℥ iii.

Sig. : Tablespoonful every hour.

**Ulcers, Epithelioma and Ulcerating Lipus.**

Dr. J. V. Shoemaker reports some excellent results in the class of cases just enumerated from the application of a saturated solution of jequirity. The experiments with this valuable drug are still being further investigated at the Philadelphia Hospital for Skin Diseases.—*The Medical Bulletin Advertiser.*

**Hemorrhoids.**

Dr. Benj. Lee, of Philadelphia, recommends the following (*Med. Times*) :

℞

Pulv. rhei., ℥ iv.

Pulv. aloes, ℥ iij.

Pulv. myrrh., ℥ ij.

Sapon. hisp., ℥ ijss.

Ol. cajeput., ʒ j.

The powders are to be rubbed together and the soap then worked in, afterward the oil. The well mixed mass is kept in tight bottles. The fresher it is, the better. Three grains of this mass make an effective pill, which is non-irritating, and may be used a long while without diminishing the susceptibility of the intestines, and often with positive benefit to the hemorrhoidal affection.—*Am. Med. Digest.*

**Neuralgia.**

I notice in the *Therapeutic Gazette* for March a request by Dr. T. B. Marchand for treatment of neuralgia, due to irritation of trigeminus nerve (fifth pair) and its branches. Let the doctor apply his medication directly on cotton in the cochlea of the ear, where the nerve lays, so very near to cutis. I have obtained the best results in most severe cases by local applications of fluid extract viburnum opulus and belladonna. My favorite formula is:

℞

Fl. ext. belladonna,
Fl. ext. viburnum opulus,
Fl. ext. gelsemium, equal parts.

M. Sig. Soak cotton in this fluid and introduce into ear as needed to relieve neuralgia in the head or toothache. Renew as needed.

S. W. M. CZARTORYSKI, M. D.

**Paralysis Agitans.**

Hammond recommends the following :

R.

Zinci Phosph., gr. ijss.
Ext. Nux Vom., gr. v.
Cons. ros. u. s. ut fiant pil. No. xxx.

Sig : One pill three times a day.

At the same time use the galvanic current on the spinal column and on the sympathetic in the limbs showing the tremors.

**Comedones (Black-head Grubs.)**

The following application is of utility :

R.

Kaoline, (potters' clay), 4 p.
Glycerine, 3 p.
Ac. Acetic., 2 p.

M. Sig. : Cover the part affected, with this paste, in the evening ; after several days of this application, most of them come out by washing with pumice stone soap.

**Whooping Cough.**

By Dr. W. C. Webb.

R.

Croton Chloral, gr. xv.
Aeth. Sulph., gtt. xx.
Pot. Brom., ʒ i.
Tinct. Bellad., gtt. xv.
Tinct Hyoscyami, gtt. xxiv.
Syr. Tolu ad, ℥ iv.

M. Sig. : One teaspoonful every 4 hours until better, then only three times a day.

**Angina Pectoris.**

Dr. Matthew Hay, demonstrator of Materia Medica in the University of Edinburgh, has used the nitrite of sodium very satisfactorily in a case of angina pectoris. So far as he is aware, this is the first case of angina pectoris in which a simple nitrite has been used. He thinks that it is as active and reliable as nitrite of amyl or nitroglycerine, at the same time possessing distinct advantages over either of these, notably in producing, in therapeutic doses, no disagreeable general effects—headache, giddiness, and even partial collapse. The objection to it is its tendency, in large doses, to produce eructations of nitrous acid gas, which, however, does not occur when small doses are administered. The formula used by Dr. Hay was : ℞ Sodii Nitrit, ℥ ss ; Aquæ. ad ℥ xij. Solv. S. Dose, one or two teaspoonfuls.—*Practitioner.*

**Irregular Action of Heart.**

Dr. Bowditch uses the following :

℞

Pulvis Digitalis, gr. x.
Pulvis Colchici Sem., gr. xx.
Sodii Bicarb., gr. xxx.

M. ft. pulvis, in chart., No. xii div.

Sig. : One powder three or four times a day.

---

**Dysmenorrhœa Mixture.**

℞

Iodide of potassium, ʒ ii.

Sulphate of iron, ʒ jss.

Distilled water, ℥ ij.

Tincture of cardamoms, ʒ iv.

Syrup of ginger, ʒ xij.

Mix ; dose, a teaspoonful three times a day, in a little water. Use two weeks before the period, for two periods.

---

**Consumptives.**

Gmeiner has employed petroleum with excellent results in consumption. In one case where iodine, quinine, chloride of ammonium and antimony did not give the patient the slightest relief, crude petroleum in its natural state caused perceptible improvement. Half a teaspoonful was given three times a day in sherry wine. In four days the pulse, that had been 120 per minute, became normal, and in less than a month the cure was complete. It is best given in capsules, as the odor is more repulsive to the patient than the taste.—*Repetit. di Chim. e Farm.* [The source of the oil is not stated, and may be important.]

---

**Preventive of the Poisonous Effects of Salicylic Acid.**

Dr. Schilling, in *Allgem. Med. Central Zeitung*, recommends the administration of ergot in conjunction with salicylic acid or quinine, to obviate the unpleasant effects of those drugs. He had observed, in a number of cases in which large doses of salicylic acid were taken, a marked congestion of the external auditory canal and membrana tympani. He was thus led to give ergot to cause a contraction of the vessels, and obtained in every case a cessation or notable diminution of tinnitus and deafness. The dose of ergot (aqueous extract) should be about one-tenth that of the salicylic acid. The antipyretic effect of the latter is not weakened by the ergot. Like favorable results were obtained by combining ergot with quinine.

**Gargle in Diphtheria.**

When the patient is old enough to gargle, the following is one of the most valuable combinations that can be employed :

℞

Acid lactic, gtt. xx to xxx.
Sps. Rectific. ℥ iiss.
Glycernæ ℥ ss.

M. S. gargle frequently.

---

**Action of Quinine.**

It is claimed that the administration of a weak tartarac acid lemonade, immediately before swallowing powder or pills of quinine, will facilitate the action of this drug. In addition to its effect in thus accelerating solution and absorption, it is said also to obviate the unpleasant gastric irritation experienced in the case of some after the administration of large doses of quinine.

---

**Vomiting of Pregnancy.**

Dr. Torino has found that during pregnancy there are often points of tenderness along the spinal column. He attributes the vomiting to spinal irritation and anæmia of the cord.

In support of this theory he adduces the satisfactory results often obtained by the administration of phosphite of zinc and nux vomica. Under the influence of these drugs the vomiting is frequently controlled and the pains of spinal tenderness disappear.—"*L.*" *Union Medicale.*

---

**Diphtheria.**

Dr. Lolli, of Trieste, uses exclusively the following mixture in the treatment of diphtheria, and in sixty cases the mortality was less than two per cent., the malady having a duration of but eight or ten days, and being but rarely propagated to the mucous membrane of the respiratory organs :

℞

Ferri sesquichlorid, 15 to 45 gr.
Acidi carbolicæ, pur., 15 to 45 gr.
Mel. rosæ, 1 ounce.
Aquæ calcis, 15 fl ounces.

The throat is swabbed with this mixture every half hour, adults using it as a gargle, and it is besides to be taken in tablespoon doses, diluted, every second hour. Of course, tonics and very nourishing food form most important adjuncts to the treatment.—*Journal Materia Medica.*

**Congestive Chills.**

Dr. Thomas Keefe, of Deming, New Mexico, states (*Medical Age*) that he has employed jaborandi with entire success in quite a number of cases of congestive chills. His *modus operandi* is to inject subcutaneously about 29 ♏ of the fluid extract of jaborandi, which is to be repeated in 20 minutes if necessary (which is seldom the case). In abont fifteen minutes profuse perspiration sets in, the chill is broken, and the patient rapidly recovers.

**Pulverized Vaccine Matter.**

An important discovery by Dr Reissner promises to do away with all scarcity of vaccine matter in the future. It offers few difficulties, and every country physician can provide himself with a practically unlimited quantity of the purest animal vaccine virus, at a nominal expense.

A calf is vaccinated with pure virus, and after five full days the pustules are taken off with a pair of forceps, and shaped with a lancet and pressed so as to obtain all the fluid lymph contained. This lymph is spread on glass plates and placed in a sulphuric acid desiccator, where it remains one or two days. It is then taken out, pulverized in a mortar, and replaced in the desiccator till wanted, when it is mixed with a little water or glycerine.

Thus prepared, the virus from one calf will vaccinate 2,000 to 3,000 persons. The calf is as well as ever in a few days, and the physician is provided with the purest of bovine virus for an indefinite period.—*Med. and Surg. Reporter*.

**Chronic Tonsillitis and Tonsillotomy.**

The London *Medical Times* of March 8, contains a brief but pointed paper condemning the wholesale removal of tonsils for chronic inflamation. The disease is peculiar to certain individuals, usuasly those of a strumous type. In many cases other lymphatic enlargements coexist. The cause of the fluctuation in size in the same person, and of the growth after removal, if to be looked for in the anatomy of the organ. It is made up entirely of lymphatic and adenoid tissue. The symptoms produced are : the peculiar expression of the face, the breathing at night, occasional deafness, and less frequently abdominal pain and capricious appetite. So long as deglutition and respiration are not interfered with, the size of tonsils is usually a matter of little importance. A strong tendency to atrophy, as age advances, exists. When tendency to hypertrophy is still present, it is almost certain to show itself after ablation. The general, not the local condition should be aimed at as the object of treatment. In most cases it is found that cod-liver oil and iron will do as good work before as after operation. Except for the one cause of local obstruction, there is no more reason for removing hypertrophied tonsils than any other enlarged lymphatic glands.—*Arch. of Pediatrics*.

**Diphtheria.**

A good deal of prominence was given last fall, says the *Sanitary Engineer*, in the papers to the occurrence of diphtheria in a family in Amsterdam, N. Y. Two children died at intervals of several mont s, and a third was taken sick. The board of health appointed a committee to investigate, which examined the house and its surroundings, and obtained a statement from the attending physician. They have recently made their report, finding that there were no bad conditions existing in or about the house sufficient to explain the appearance of the disease, and they conclude that it came from a cat which was fondled by the child which first fell ill. This cat was found at the time to have a swollen throat and to be suffering from a discharge from the mouth and nostrils. It died a few days afterward. Three days after the death of the cat the child fell sick with malignant diphtheria and died in about a week. During its illness it played with a doll which was afterward given to a younger child, as it was supposed to have been properly fumigated with sulphurfumes. This child, shortly after being allowed to play with the doll, also fell ill of diphtheria and died. The third child also played with the doll and fell ill, but recovered. The board of health, therefore, traces the re-appearance of the disease in the family after the death of the first child to the doll.

**Cremation in Japan.**

Cremation is becoming very general in Japan and at the present time the number of bodies disposed of in this way is about 9,000 each year. The cremation chamber is built with stone and cement, and has a very tall chimney which makes it look like a factory. In the vestibule are a number of red earthen wares, urns and small shovels, which the relatives of the deceased purchase to collect the ashes of the defunct. Behind this vestibule are four chambers, the largest of which is decorated with granite columns. The bodies are buried in this chamber, at the rate of one year (?) (about 3s. 6d.) each, but the families who wish to have a private cremation have to pay five years (?). After the funeral ceremony has been held at the house of the defunct, the body is moved to the place of cremation, and watched over by a priest until eight in the evening, when the fire is lighted, and burns all night. At six in the morning the ashes are collected and placed in an urn, which is interred, often with much pomp, at the cemetery. No unpleasant odor is emitted, either during or after the operation, and this is attributed to the high chimney. The simplicity of this process is remarkable, and it answers the purpose as well as the more complicated and expensive methods resorted to in Europe. The building itself is hedged in by fences of bamboo-canes and red camellias.—*London Sanitary Record.*

---

**Cholera Remedy.**

THE *Chemist and Druggist* of July has the following :

None will be surprised that the current literature, medical or otherwise, should be filled with suggestions for the treatment of cholera. Amongst others, Dr. Tison draws attention to a remedy which obtained a certain celebrity in Paris during two previous visits of this epidemic. It was introduced by Dr. Roux both in 1849 and 1852, and appears in his hands to have been successful. Other physicians of note also were fortunate in its use. The formula is as follows : Dissolve 10 grains of washed sublimed sulphur in rectified sulphuric ether, aiding the solution, if necessary, by gentle heat ; immersion of the bottle in warm water is sufficient. Ether dissolves $\frac{1}{80}$ of its weight of sulphur ; rectified spirit only $\frac{1}{100}$; consequently highly rectified ether should be selected ; 25 drops to 30 are added to half a wine-glass of sweetened water ; seltzer water sufficient to fill the wine-glass completing the mixture, which is then to be given in divided doses. Simple as the preparation seems, it is stated to have proved effectual. M. Boutigny, in an inaugural thesis in 1872, strongly supported this statement, declaring that he had obtained signal success by means of the ethereal solution of sulphur. A strange circumstance is related by the same authority. A patient, evincing dislike to ether as a remedy, had administered to him as a substitute an opium and bismuth preparation, when the symptoms which had yielded to the ether treatment immediately reappeared.

---

**Intermittent and Remittent Fevers.**

To those physicians who, like myself, have to dispense their own prescriptions, and for a great many of them who receive no pecuniary acknowledgment (the cost of the drugs thus given away amounts to a good sum in the course of a year, especially in a locality infected with malarial diseases, as the greater part of our State is), for the benefit of all, especially those who furnish their own medicines, I subjoin two prescriptions. The first will interrupt any acute attack of intermittent or remittent fever, and the second will prevent any relapse of either, and at the same time will act as a good and efficient tonic in the anæmic condition always found in patients laboring under chronic intermittent fever. The beauty of these prescriptions is their certainty and low cost, and I can safely promise any physician they will save him many a dollar in the course of a year, if he furnishes his own medicines.

℞

Sul. cinchonia, ss ℥.

Capsicum, j ʒ.

Ext. colocynth comp., j ʒ.

Pulv. ipeca. comp., ij ʒ.

M. Of this take 40 grains div. in Chart. No. x., give one every two hours during paroxysms.

Should the tongue be much coated, give a vegetable cathartic, after chill has stopped, in intermittent, but administer cathartic at once in remittent.

℞

Arsenious acid, gr. iv.

Sul. cinchonia, gr. lxxx.

Iron, reduced, ij ʒ.

Solid ext. gentian, ij ʒ.

M. Ft. Pillulæ No. lxxx.

Sig. : One after each meal.—*A. T. Levick, M. D., of Woodville, M. O., in Medical Brief.*

---

**Treatment of Tonsillitis.**

Dr. J. Solis Cohen, in *Medical News*, gives the following treatment:

In simple inflammatory tonsillitis, take two fluid drachms each of the ammon. tinct. of guiac. and the comp. tinct. of cinchona, mix with six fluid drachms of clarified honey and shake together until the sides of the vessel are well coated; add gradually a solution of eighty grains of chlorate of potassium in four ounces of water, shaking meanwhile. This is to be used as a gargle every one-half to three hours. Relief is usually experienced within a few hours and recovery is prompt. A saline cathartic may accompany the use of the gargle. None of the cases seem to have suppurated, and if seen within the first twenty-four hours such accidents are very unlikely.

2. In rheumatic or constitutional tonsillitis (characterized by intense pain in swallowing, causing great accumulation of saliva from unwillingness to swallow, with slight, perhaps no congestion of throat and subsequent fever; one or both tonsils becoming enlarged after some hours as the febrile symptoms decline, and muscular or joint rheumatism sometimes developing later) after a saline cathartic, give the following in tablespoonful doses every two hours:

R

Sodii salicylate, ʒ ij,

Ol. gaultheriæ, ♏ j,

Liq. ammon. citrat,

Syrup simp. aa ℥ ij.

Lengthening the intervals as the pain subsides. Pieces of ice or guiac. gargle promote comfort, and the stiff neck is best relieved by Faradization. Salicylate of quinine or cinchonidine may be substituted for the above if a tonic be required, in five grain doses every four or six hours.

---

**Croup—Diphtheria.**

In reading a communication from a correspondent in last week's *Reporter* in reference to the treatment of croup by lime inhalations, I was reminded of many cases I had treated in this way years ago with marked success. My attention was first called to this mode of treatment by seeing a notice of it in Vol. XIII of this journal, 1865, page 171. Those that may feel an interest will do well to refer to the above number of the *Reporter*. I will quote a few sentences. After stating that a German by the name of M. Kuchenmeister had found that "diphtheritic membranes are rapidly dissolved in lime water," the *British Foreign Medical and Chirurgical Review* says that some pseudo-membranous exudations of considerable extent and thickness were placed in a small glass of lime water, and in the space of from ten to fifteen minutes they disappeared, leaving only a very slight sediment at the bottom of the glass. It having been my lot heretofore to have been called to many cases of croup, many of which had run on rapidly to a fatal termination, I was therefore particularly anxious to find a remedy that would resist the great fatality of this disease. Since adopting the lime inhalation treatment my success has been almost uniform. But the lime inhalation will only answer where the deposit is that of true croup. In the croupous form of diphtheria, or diphtheric croup, it has no effect whatever, as many trials of it, at different times, have fully satisfied me. But like lime in the treatment of croup, so sulphurous acid and sublimed sulphur seemed to be the most reliable remedies in removing the deposit of diphtheria. Sublimed sulphur, as it comes to us, is always acid, and if we expose it to the air for a time, it becomes still more so. This, blown into the fauces, or placed dry upon the tongue, I have seen remove the membrane in a very short time. So it would seem that an acid was indicated in the treatment of croup. That the deposit of croup and that of diphtheria are widely different, I am fully satisfied. The croupous form of diphtheria often so simulates true croup that at times a correct diagnosis becomes difficult. But it is very necessary that an early discrimination should be made, as much valuable time may be lost in letting these formidable complaints run on their natural course.

In the spring of 1857, several cases of membranous croup occurred in my circuit. Those that were seen early, it was found that small and repeated doses of calomel was the most reliable remedy, but the case had to be seen early. Two cases proved fatal in one family in the spring of that year. With the assistance of Dr. S., then of Mt. Holly, I removed a section of the trachea of one of these fatal cases. We found the trachea so filled with deposit that a knitting-needle would scarcely pass through. The specimen was exhibited at a meeting of our Medical Society, and elicited much interest. It finally passed out of my hands, and I think was placed in the museum of the Jefferson College. None of these cases, I think, had any deposit in the fauces, or above the epiglottis.—Wm. L. Martin, M. D., in *Med. and Surg. Reporter*.

**Formula Used in New York Hospitals.**

1. ITCH. The patients are thoroughly annointed in the affected places with the compound naphthol ointment (10 per cent., see below) without having previously been washed, and then dusted with starch. A single, but thorough application is enough.

In the case of children, the amount of napthol is to be reduced one-half.

2. ECZEMA. Only in the squamous condition has a weak (½ to 1 per cent.) naphthol ointment given satisfactory results. On the other hand, it is an excellent application in the special form of eczema accompanying scabies, prurigo and ichthyosis.

3. PRURIGO. Kaposi treats all cases of prurigo with a 5 per cent. naphthol salve, to be rubbed in once in the evening, and dusted over with starch. The success is reported to be all that could be desired. In cases of hyperidrosis of the hands or soles of the feet, or axilla, a 5 per cent. alcoholic solution, applied once or twice daily, and the place dusted over with starch (or with a mixture of 92 parts of starch and 8 parts of naphthol, was found to be almost always effective.

The following combinations have been recommended by Kaposi and others :

1. COMPOUND NAPHTHOL OINTMENT.

*Itch Ointment.*

℞

Naphthol, 10 parts.

Cretæ albæ pulv., 10 parts.

Saponis viridis, 40 parts.

Adipis, 40 parts.

2. NAPHTHOL OIL.

℞

Naphthol, 1.0 part.

Ol. olivæ,

(or Ol. morrhuæ, or Ol. amygdalæ, etc.) 99.0 part.

(In eczema crustosum, pediculosis capillitii, herpes ton surans, favus.)

3. NAPHTHOL SOLUTION.

℞

Naphthol, 1 0 part.
Glycerini, 5.0 part.
Alcohol, 94.0 parts.

(In eczema marginatum, to be brushed on during five to eight days, until a light-brown, smooth crust is formed.)

4. NAPHTHOL APPLICATION, recommended in eczema marginatum :

℞

Naphthol, 1.0 part.
Lac. sulphur, 5.0 part.
Glycerini, 2.5 part.
Tinct. sapon vir., 20.0 part.
Alcoholis, 100.0 parts.

5. NAPHTHOL APPLICATION, to be used in acne vulgaris and rosacea, sycosis, and lupus erythematosus :

℞

Naphthol, 1.0 part.
Lac. sulphur, 10.0 part.
Balsam Peruv., 2.0 part.
Tr. saponis vir., 25.0 part.
Spir. vini gall., 50.0 part.

6. NAPHTHOL SOLUTION, for hyperidrosis of the hands and feet :

℞

Naphtol, 5.0 parts.
Glycerini, 10 0 parts.
Alcoholis, 100.0 parts.

It will be of advantage to give, in this place, the more prominent characteristics and properties of naphtol :

Pure naphtol is in form of white, shining needles. As usually met with, it is either in form of whitish, or yellowish-white, or grayish, lustrous crystaline grains. The crude substance occurs in deep violet-brown or lighter lumps easily reducible to powder. When pure it is nearly (or entirely ?) odorless ; it has a sharp, burning taste, and its powder, when inhaled up into the nostrils, excites violent sneezing. It is but slightly soluble in boiling water, but very easily in alcohol, ether, chloroform, benzol, oils and fats. The saturated aqueous solution at 25 degrees C. contains about one part of naphthol in 550. When gently heated, it sublimes, and it may be distilled with steam. This property should be borne in mind, when making a solution of it in hot water.

A large quantity of solution (say a barrel) is best made by dissolving the requisite amount of naphthol in about twice its weight of alcohol ; then, having put the necessary weight of hot water in the barrel, add the alcoholic solution gradually, stirring or shaking after each addition, until all is added and the naphthol is dissolved. Then allow to cool.

